# MSP-RON Pathway: Potential Regulator of Inflammation and Innate Immunity

**DOI:** 10.3389/fimmu.2020.569082

**Published:** 2020-10-07

**Authors:** Lingtong Huang, Xueling Fang, Danrong Shi, Shuhao Yao, Weifang Wu, Qiang Fang, Hangping Yao

**Affiliations:** ^1^Department of Critical Care Units, The First Affiliated Hospital, Zhejiang University School of Medicine, Hangzhou, China; ^2^State Key Laboratory for Diagnosis & Treatment of Infectious Diseases, National Clinical Research Center for Infectious Diseases, Collaborative Innovation Center for Diagnosis and Treatment of Infectious Diseases, The First Affiliated Hospital, Zhejiang University School of Medicine, Hangzhou, China; ^3^Department of Stormotologry, Wenzhou Medical University Renji College, Wenzhou, China

**Keywords:** MSP, RON, macrophage, innate immunity, inflammation, autoimmune disease

## Abstract

Macrophage-stimulating protein (MSP), a soluble protein mainly synthesized by the liver, is the only known ligand for recepteur d'origine nantais (RON), which is a member of the MET proto-oncogene family. Recent studies show that the MSP-RON signaling pathway not only was important in tumor behavior but also participates in the occurrence or development of many immune system diseases. Activation of RON in macrophages results in the inhibition of nitric oxide synthesis as well as lipopolysaccharide (LPS)-induced inflammatory response. MSP-RON is also associated with chronic inflammatory responses, especially chronic liver inflammation, and might serve as a novel regulator of inflammation, which may affect the metabolism in the body. Another study provided evidence of the relationship between MSP-RON and autoimmune diseases, suggesting a potential role for MSP-RON in the development of drugs for autoimmune diseases. Moreover, MSP-RON plays an important role in maintaining the stability of the tissue microenvironment and contributes to immune escape in the tumor immune microenvironment. Here, we summarize the role of MSP-RON in immunity, based on recent findings, and lay the foundation for further research.

## Highlights

- This review focuses mainly on the role of the MSP-RON signaling axis in inflammation and innate immunity.
- The objectives of this study are to summarize the major findings in this research field, to establish the importance of MSP-RON signaling in inflammation and innate immunity, and to provide a foundation for future experiments and studies.
- This review covers four topics: an introduction to MSP and RON, the biological functions of RON in innate immunity, the role of MSP-RON in acute and chronic inflammation, and aberrant RON signaling in autoimmune diseases and its influence on the tissue microenvironment.
- Abnormal activation or inhibition of the MSP-RON signaling axis is a potential strategy for drug development.

## Introduction

**Figure 1:**
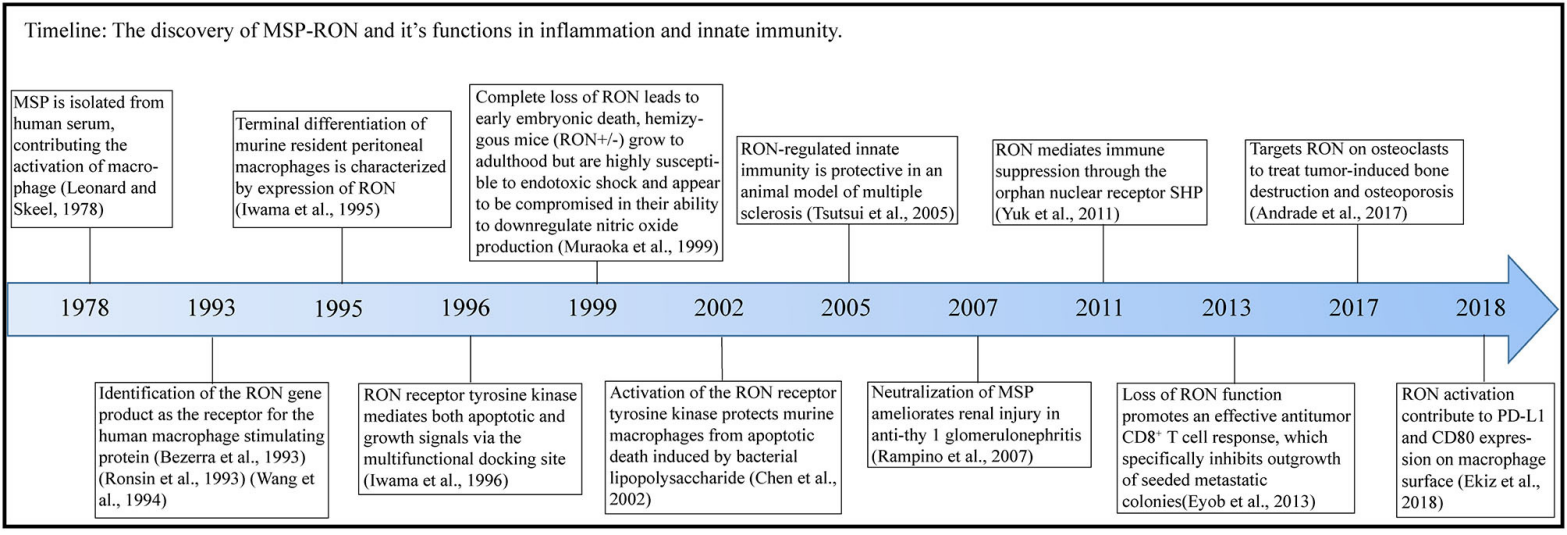
Timeline. In this figure, we illustrate the timeline of MSP-RON discovery and its role in inflammation and innate immunity.

Macrophage-stimulating protein (MSP) was first purified in 1978 (1) and is secreted by the liver and released into the blood (2) [Figure 1 (Timeline)]. Pro-MSP, with a molecular weight of 78 kDa, is cleaved by various enzymatic systems to form a biologically active MSP, consisting of α and β chains, and characterized by the presence of a highly conserved Kringle domain (3). The β chain of MSP binds to the transmembrane protein RON (recepteur d'origine nantais, also known as MST1R or CD136w) but has no biological activity alone; only the complete mature MSP exhibits biological activity (4). MSP is of great interest to researchers due to its function in promoting migration and phagocytosis by activating macrophages (1, 3) and regulating the inflammatory response of macrophages (5). RON is a member of the MET family of receptor tyrosine kinases (RTKs) and was isolated from a cDNA library in 1993 (6) and named as stem cell-derived tyrosine kinase (STK) in mice (7, 8). MSP was subsequently shown to be a ligand for the RON receptor (9).

MSP can bind to RON on the surface of tissue-resident macrophages, thereby inhibiting inflammatory response (10). MSP-RON is also associated with autoimmune diseases, such as inflammatory bowel disease (11) and multiple sclerosis (MS) (12). In addition, RON has been shown to be abnormally expressed on many cancer cells, thus promoting tumor migration and proliferation (13–15). Phosphorylation of Tyr1238 and Tyr1239 of RON activates ERK and PI3K/AKT and subsequently mediates tumor proliferation and survival (13). Tyrosine kinase inhibitors and some monoclonal antibodies of RON receptor have been shown to inhibit the growth and proliferation of various tumors such as triple-negative breast cancer and pancreatic ductal adenocarcinoma (14, 15).

In recent years, more and more studies show that the MSP-RON signaling pathway plays an important role in immunity and various immune diseases. This article reviews the role of MSP-RON in the immune system, which participates in acute disease, chronic disease, and tumor immune escape, and highlights few key research questions that need to be addressed.

## RTKs and Activation of RON

RTKs play an important role in the body. There are similar intracellular segments in different receptor families, which regulate similar signaling pathways and exhibit similar functions, including activation of the PI3/Akt and Erk1/2 signaling pathways to promote cell proliferation, differentiation, migration, and cell cycle progression (16). The RTKs, TYRO3, AXL (also known as UFO), and MERTK exhibit immune effects similar to those of MSP-RON activation, including promoting M2 differentiation of macrophages, reducing the production of pro-inflammatory factors, and antagonizing the Toll-like receptor downstream pathway (17).

MET, a subfamily of RTKs, consists of two members, MET and RON. Their extracellular SEMA domain is the key to identify with other RTKs (16). MET and RON have similar but not identical SEMA domains, which may account for their structural similarity, but different biological functions (18). Crystal structure analysis confirmed that one MSP molecule interacts with the SEMA domains of two RON molecules to cause receptor dimerization and activation, thereby activating the downstream Akt and Erk1/2 pathways (13). Further, in a study by Angeloni et al., addition of soluble RON-related SEMA proteins or SEMA + PSI proteins during macrophage culture, to competitively bind MSP, weakened the binding of MSP to RON receptors on macrophages and inhibited the activation of downstream pathways, indicating the specific binding of MSP to SEMA domain and suggesting that soluble RON-related SEMA proteins may be used for blocking the MSP-RON pathway (19).

## MSP-RON Involves in Immune System

MSP can activate RON in tissue-resident macrophages in the liver (20), lung (21), bone (22, 23), and brain (24) as well as tumor-associated macrophages (25) to regulate macrophage phagocytosis (26), migration (27, 28), and other functions. MSP-RON also promotes epithelial cell proliferation following injury (29), participates in tissue repair (30), and promotes lung ciliary movement (29, 31). Therefore, it also plays a role in non-specific immunity.

RON expression can be used as a marker of terminal differentiation of resident macrophages (32). Cell behavioral changes caused by pathway activation are different in different cells. The most typical behavioral change in macrophages caused by the activation of MSP-RON is the activation of downstream PI3/Akt, which promotes phagocytosis of complement C3bi-coated red cells through CR3 and ICAM-1; besides, treatment with the PI3K inhibitors, wortmannin and LY294002, can inhibit this effect (22). MSP, at an optimal dose of 0.2 nM, can also induce the migration of resident peritoneal macrophages in a short time (27). MSP-RON-induced activation of osteoclasts, a special type of bone tissue-resident macrophages, enhances osteoclast bone resorption capacity (22) but does not enhance proliferation (33). During breast cancer bone metastasis, because breast cancer cells secrete more MSP, the MSP-RON pathway is activated in the osteoclasts, resulting in bone destruction; however, treatment with the RON inhibitor BMS777607/ASLAN002 can reduce bone destruction and decrease the expression of osteolytic markers in patients with breast cancer (23). While RON is highly expressed in the lung ciliated epithelial cells, MSP is found in high concentration in the bronchial epithelial cells, and activation of MSP-RON increases the ciliary beat frequency (29). The possible reason is the colocalization and physical binding of receptor for hyaluronic acid-mediated motility and RON, and functional changes in RON and ciliary beat frequency occur by decomposing high-molecular-weight hyaluronic acid into low-molecular-weight hyaluronic acid fragments and combining with receptor for hyaluronic acid-mediated motility (31), which may contribute to non-specific immunity.

When infected with Epstein–Barr virus, latent membrane protein 1, an oncoprotein associated with Epstein–Barr virus, can promote the binding of NF-κB to the RON promoter, inducing the expression of RON in B cells and promoting tumor cell proliferation (34). MSP-RON activation is also sufficient to replace erythropoietin for erythroid cell proliferation; induce the phosphorylation of Gab1, MAPK, and PKB; and enhance the proliferation of erythroid progenitor cells (35). However, it seems that the activation of the MSP-RON pathway varies in different cells. In STK/RON-transfected Ba/F3 pro-B cells, MSP stimulation promoted proliferation, while in STK/RON-transfected mouse erythroleukemia cells, it resulted in apoptosis (36), which is inconsistent with the previous finding of the anti-apoptotic effect of MSP-RON activation through PI3/Akt and Erk1/2 (37, 38).

### The Regulation of Acute Inflammation

**Figure 2:**
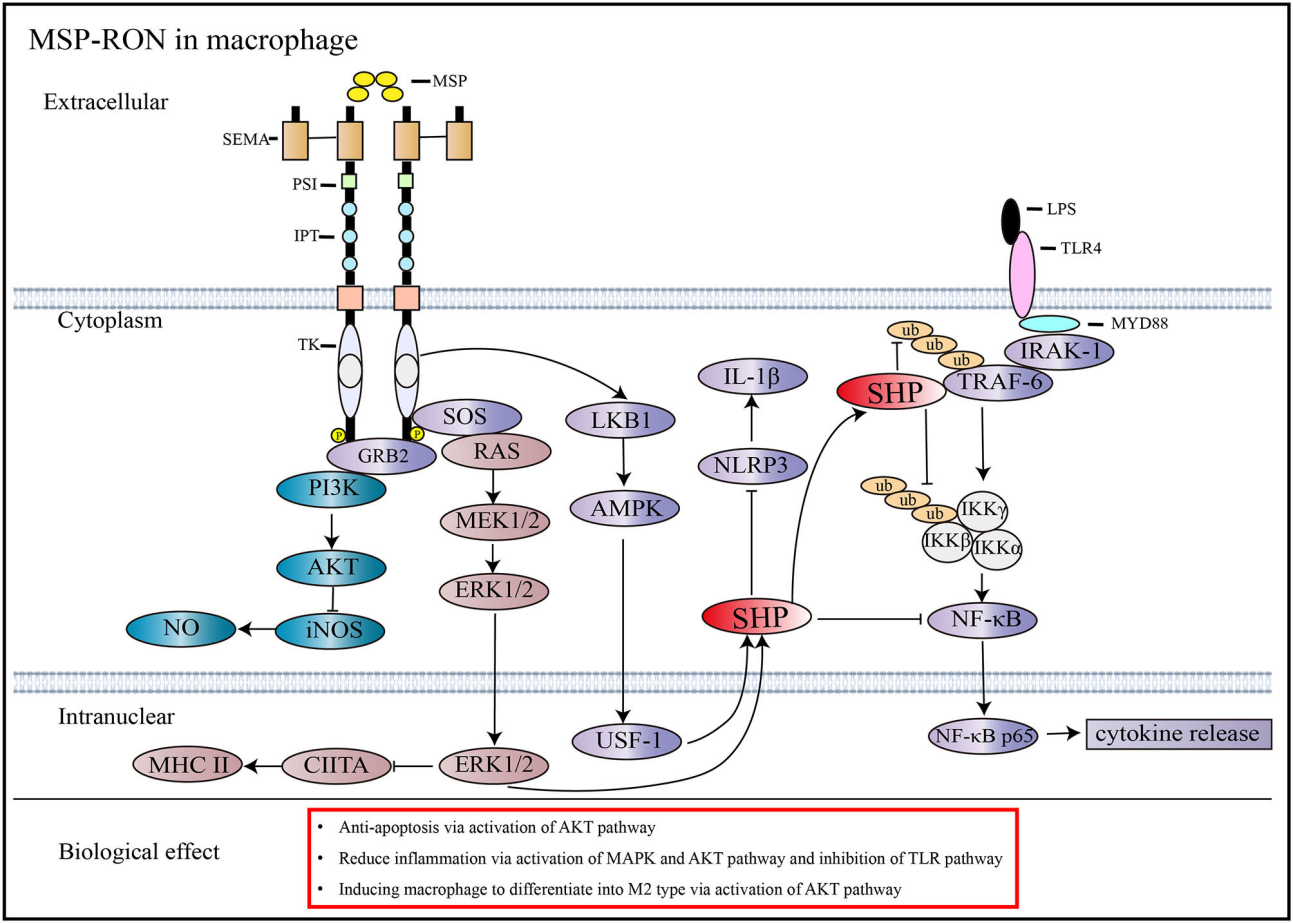
Signaling pathways activated by MSP and RON. MSP induces RON dimerization and activates downstream pathways (13). In macrophages, the activation of RON inhibits iNOS through the AKT pathway and reduces NO synthesis (10). RON activation stimulates SHP expression through the MAPK and AMPK pathways, which can antagonize the TLR4 pathway by inhibiting TRAF6 ubiquitination and preventing NF-κB from entering the nucleus, thus reducing cytokine production (39). Besides, increased expression of SHP through activation of the MSP-RON pathway can inhibit NLRP3 inflammasome activation, thereby inhibiting cleavage of pro-IL-1β into activated IL-1β (40). MSP-RON activation also inhibits CIITA transcription and, thus, MHC II expression via the MAPK pathway (41).

During acute inflammation, MSP-RON can activate the PI3/Akt pathway, which is necessary to inhibit the expression of nitric oxide synthase in macrophages (10). MSP-RON can also activate the LKB1-AMPK pathway to induce the orphan nuclear receptor small heterodimer partner (SHP) transcription, thereby inhibiting TRAF6 polyubiquitination and suppressing Toll-like receptor (TLR) signaling (39) (Figure 2). In addition, increase in SHP expression inhibits assembly of NLRP3 inflammasome and maturation of interleukin (IL)-1β (40) (Figure 2). Studies have shown that nitric oxide production induced by lipopolysaccharide (LPS) strongly inhibits the mRNA and protein expression of RON in mouse peritoneal macrophages within 72 h, and the combined use of tumor necrosis factor-α and interferon-γ also produces similar results; this effect can be antagonized by stimulation with MSP or transforming growth factor-β (10). However, some studies have obtained different results. Treatment of mice with 3 mg/kg LPS was shown to induce RON mRNA and protein expression in liver macrophages and endothelial cells (42). Further, co-stimulation of FVB mice with LPS and MSP increased the expression of RON more significantly than stimulation with LPS alone (43).

Activation of the MSP-RON pathway antagonizes LPS-induced inflammatory factor production (20, 42) and reduces LPS-induced peritoneal macrophage apoptosis (55). Moreover, the lack of RON receptors impairs the anti-inflammatory ability of mice after LPS stimulation (56, 57), with increased liver (20) and lung tissue damage (21, 58, 59). A previous study showed that, in a classic mouse model of sepsis, the survival time of RON knockout mice was significantly reduced, and colony formation in systemic organs was significantly increased, accompanied by increased liver damage (44) (Table 1). This may be because RON knockout resulted in reduced production of IL-6, macrophage inflammatory protein-2, and monocyte chemotactic protein-1, which are important for neutrophil mobilization by macrophages (44). MSP-RON activation in macrophages can inhibit IL-12p40 expression, which may lead to activation of NK cells and γδT cells (60, 61). Previously, RON knockout mice were shown to display significantly increased interferon-γ production after LPS stimulation (41). RON activation can inhibit the expression of CIITA through the activation of Erk1/2, thus reducing the expression of MHC II in macrophages as well as the ability to activate T cells (41) (Figure 2). This indicates that the MSP-RON pathway not only acts on macrophages but also exerts a wide range of anti-inflammatory effects through signal transmission between cells. In a nickel-induced lung injury model, RON knockout were shown to exhibit enhanced inflammatory response and a significantly shorter survival time (45), suggesting that MSP-RON not only has an antagonistic effect on LPS-induced production of inflammatory mediators but also has protective effects on inflammatory responses induced by other substances. Interestingly, in FVB mice with M2 susceptibility, the TLR4 pathway is more significantly antagonized than that in M1-prone C57/B6 mice (43), thus adding complexity to the understanding of the role of MSP-RON in experimental animals and populations of different genetic backgrounds. The activation of MSP-RON in human lung macrophages, especially in the alveolar macrophages of smokers, shows dual anti- and pro-inflammatory effects (62). However, further research is required to clarify the role of MSP-RON in human tissue-resident macrophages.

**Table 1:** MSP-RON with clinical diseases.

**Classification and diseases**	**Details**
**Acute inflammation**	
Sepsis	The survival time of RON knockout mice reduced with increased liver damage in a sepsis mouse model (44)
Acute lung injury	RON knockout was shown to exhibit enhanced inflammatory response and a significantly shorter survival time through macrophage in nickel-induced lung injury model (45)
**Chronic inflammation**	
Non-alcoholic steatohepatitis	MSP-RON antagonizes inflammatory response induced by oxidized low-density lipoproteins and LPS via activating the AMPK signaling pathway (46). Activation of RON improves diet-induced fibrosis in non-alcoholic fatty liver mice, and RON knockout mice exhibit higher levels of inflammatory cell infiltration, collagen, ECM remodeling proteins, and profibrotic cytokine expression (47)
Obesity	MSP-RON reduces inflammation, increases tissue repair capacity, and induces macrophages to switch to M2 phenotype in mouse obesity model (48); RON knockout resulted in increased serum levels of inflammatory factors in mice with high-fat diet (49)
**Autoimmune diseases**	
Ulcerative colitis	The expression of RON in intestinal mucosal epithelial cells is higher than that in healthy patients (50, 51)
Crohn's disease	Genome-wide association studies shows that SNP of rs3197999 in the MSP gene is associated with Crohn's disease (11)
Primary sclerosing cholangitis	Genomics study of PSC patients showed that PSC is related to SNP rs3197999 (MSPβR689C) (52)
Multiple sclerosis	The expression and synthesis of RON were significantly reduced in patients with MS and after RON knockout, central nervous system inflammation significantly increased in experimental autoimmune encephalomyelitis animal models (12), but SNP rs3197999 (MSPβR689C) is not associated with MS (53)
Anti-Thy-1 nephritis	Promoting mesangial cell proliferation and blood mononuclear cell infiltration, besides antagonizing MSP, could significantly improve glomerular damage and mesangial proliferation (54).

### The Regulation of Chronic Inflammation

MSP-RON also plays an important role in chronic inflammation. Non-alcoholic steatohepatitis is a liver inflammatory disease caused by continuous stimulation with low-density lipoproteins and LPS (63). In an *in vitro* model, MSP-RON was shown to antagonize inflammatory response in mouse liver primary cells and bone marrow-derived macrophages induced by oxidized low-density lipoproteins and LPS via activating the AMPK signaling pathway (46). Moreover, activation of RON has been shown to improve diet-induced fibrosis in non-alcoholic fatty liver mice, and RON knockout mice exhibit higher levels of inflammatory cell infiltration, collagen, ECM remodeling proteins, and profibrotic cytokine expression (47). However, a recent study showed opposite results. In non-alcoholic steatohepatitis mice treated with 500 ng/day of MSP by micropump infusion, liver inflammation was enhanced with increased expression of tumor necrosis factor-α, IL-1β, CCL2, etc. (64). This study did not report the MSP dose that penetrates into the liver, which may affect the conclusions of the experiment. Similarly, MSP-RON also plays an important role in obesity-induced chronic inflammation. In a mouse obesity model, MSP-RON was shown to reduce inflammation, increase tissue repair capacity, and induce macrophages to switch to an M2 phenotype, characterized by increased arginase 1 (Agr1) expression (48). This is in accordance with the results of other studies, which showed that activation of MSP-RON promotes differentiation of macrophages into the M2 phenotype associated with tissue repair, while at the same time weakening macrophage differentiation toward the pro-inflammatory M1 phenotype (65, 66).

A similar study by Stuart et al. showed that RON knockout resulted in increased serum levels of inflammatory factors in mice on a high-fat diet; however, this study also showed that activation of RON leads to increased fat synthesis in the white adipose tissue, which increases the production of pro-inflammatory factors (49). This indicates that the MSP-RON pathway is involved in adipogenesis. At present, several studies are focused on the role of MSP-RON in chronic inflammation, especially chronic inflammation related to energy metabolism. As described above, the MSP-RON-AMPK-SHP axis is important in suppressing inflammation caused by TLR activation (39, 40), and SHP can significantly inhibit liver gluconeogenesis (67), which may link MSP-RON with acute inflammation, chronic inflammation, and energy metabolism. Similarly, phosphorylation of RON has been shown to activate the PI3K-Akt-mTOR pathway in tumor cells (13), which further links MSP-RON to energy metabolism and inflammation since mTOR is a key regulator of energy metabolism and mediates immune suppression (68).

### Association of MSP-RON With Autoimmune Diseases

MSP-RON also plays an important role in autoimmune diseases. RON receptors are widely distributed in the gastrointestinal tract, and in patients with ulcerative colitis, the expression of RON in intestinal mucosal epithelial cells is higher than that in healthy patients (50, 51). Genome-wide association studies have shown that single nucleotide polymorphism (SNP) of rs3197999 in the MSP gene is closely associated with Crohn's disease (11). A previous research showed that the mismatch of SNP rs3197999 in the MSP gene (MSPβR689C) decreased the binding capacity of MSPβ to RON by 10-fold, which seriously affects the MSP-RON pathway, resulting in the occurrence of inflammatory bowel disease (IBD) (69). However, another study showed that the mismatch of R689C in the MSP protein does not affect its ability to bind to the SEMA domain of RON, but the serum level of MSP is regulated by SNP rs3197999 (MSPβR689C), which causes a decrease in serum MSP concentration, and this may be one of the mechanisms of IBD (70). Patients with primary sclerosing cholangitis (PSC) often have IBD, and a large sample genomics study of PSC showed that it is most closely related to SNP rs3197999 (MSPβR689C) (52). Through genomics and proteomics, researchers have linked the MSP-RON pathway with autoimmune diseases, such as IBD and PSC, but more research is needed to explore its pathogenesis and develop strategies for the treatment of IBD and PSC. The MSP-RON pathway has also been shown to be associated with MS. The expression and synthesis of RON were significantly reduced in patients with MS, in animal models of MS, and after RON knockout, and central nervous system inflammation in experimental autoimmune encephalomyelitis animal models significantly increased with nerve demyelination and axonal injury (12). Meanwhile, the expression of c-Cbl, a negative regulator of RON (71), increases during the onset of MS (12). However, subsequent studies have shown that SNP rs3197999 (MSPβR689C) is not associated with MS (53). Hence, more research is needed to elucidate the role of MSP-RON in the pathogenesis and prognosis of MS. Previous studies have also demonstrated a pathogenic role of the MSP-RON pathway in anti-Thy-1 nephritis, including promoting mesangial cell proliferation and blood mononuclear cell infiltration and antagonizing MSP, which can significantly improve glomerular damage and mesangial proliferation (54). Similarly, in a sickle cell disease model, elevated levels of protease membrane type serine protease 1, which can cleave MSP precursors, were reported in monocytes exposed to hemolysis and hypoxia, resulting in abnormal activation of the MSP-RON pathway in renal endothelial cells. This led to renal injury, increasing glomerular permeability. However, treatment with the RON-specific inhibitor BMS777607 significantly reduced glomerular endothelial damage and non-M1 macrophage infiltration in sickle cell disease mice (72).

The role of RON in autoimmune diseases is still not well-studied. Abnormal activation of MSP-RON signaling leads to the occurrence of autoimmune diseases. However, more research is needed to explore the exact role of the MSP-RON pathway in autoimmune diseases.

### Association Between Tissue Microenvironment and MSP-RON

MSP-RON pathway plays an important role in tissue microenvironment, especially in tumor immune microenvironment. A study by Eyob et al. showed that the loss of RON receptors in mice significantly inhibits breast cancer metastasis by enhancing their anti-tumor capacity and increasing the number of CD8^+^ T cells (61). RON activation upregulates Fos by activating MAPK, which binds to the Arg1 promoter AP-1 to induce the expression of Arg1, while Arg1 expression in tumor-associated macrophages is significantly reduced in RON knockout mice (25). Moreover, a previous study demonstrated a high number of CD8^+^ T cells and M1 macrophages and lesser numbers of M2 macrophages in the tumor microenvironment of MSP-deficient mice, which exhibit high tumor killing ability (73). Notably, in a recent study, the activation of MSP-RON in tumor-associated macrophages significantly increased the expression of CD80 and PD-L1, and the combination of anti-RON and anti-CTLA therapy significantly inhibited tumor growth in mice (74). Taken together, previous findings suggest that activation of MSP-RON plays an immunosuppressive role in the tumor microenvironment, and inhibition of MSP-RON activation may serve as a potential strategy for anti-tumor therapy.

## Conclusion

The MSP-RON pathway mediates inflammatory response in the body. Abnormalities in the MSP-RON pathway lead to the occurrence of autoimmune diseases, and excessive activation of MSP-RON promotes tumor progression. However, we rarely see the use of clinical samples to detect the role of the MSP-RON signaling pathway in the immune system and certain questions need to be addressed. The extent of MSP-RON activation in immunity and its impact on the disease, as well as the clinical potential of MSP-RON signaling need to be further evaluated. In addition, the molecular mechanisms underlying MSP-RON signaling in autoimmune diseases need to be clarified. Further, verification of the effectiveness of therapeutic drugs in disease treatment is also necessary. Clarification of these questions will contribute to evaluate the exact role of MSP-RON in innate immunity and evaluate its potential for clinical application.

## Author Contributions

LH and XF performed the literature search, drafted the manuscript, and prepared the figures. LH, XF, DS, SY, and WW helped perform revisions and critically discussed the manuscript. QF and HY designed, supervised, and critically reviewed the final manuscript. All authors contributed to the article and approved the submitted version.

## Conflict of Interest

The authors declare that the research was conducted in the absence of any commercial or financial relationships that could be construed as a potential conflict of interest.

## Abbreviations

MSP: macrophage-stimulating protein
RON: recepteur d'origine nantais
STK: stem cell-derived tyrosine kinase
RTK: tyrosine kinase receptor
SHP: small heterodimer partner
TLR: toll-like receptor
SNP: single nucleotide polymorphism
Arg1: arginase 1
IBD: inflammation bowel disease
PSC: primary sclerosing cholangitis
MS: multiple sclerosis.
